# Circulating immune complexes contain citrullinated fibrinogen in rheumatoid arthritis

**DOI:** 10.1186/ar2478

**Published:** 2008-08-18

**Authors:** Xiaoyan Zhao, Nwora Lance Okeke, Orr Sharpe, Franak M Batliwalla, Annette T Lee, Peggy P Ho, Beren H Tomooka, Peter K Gregersen, William H Robinson

**Affiliations:** 1 GRECC, VA Palo Alto Health Care System, 3801 Miranda Avenue, Palo Alto, CA 94304, USA; 2 Department of Medicine, Division of Immunology and Rheumatology, Stanford University School of Medicine, 269 Campus Drive West, Stanford, CA 94305, USA; 3 Robert S. Boas Center for Genomics and Human Genetics, Feinstein Institute for Medical Research, 350 Community Drive, Manhasset, NY 11030, USA; 4 Department of Neurology and Neurological Sciences, Stanford University School of Medicine, 269 Campus Drive West, Stanford, CA 94305, USA

## Abstract

**Introduction:**

There is increasing evidence that autoantibodies and immune complexes (ICs) contribute to synovitis in rheumatoid arthritis (RA), yet the autoantigens incorporated in ICs in RA remain incompletely characterised.

**Methods:**

We used the C1q protein to capture ICs from plasma derived from human RA and control patients. Antibodies specific for immunoglobulin were used to detect ICs, and fibrinogen antibodies were used to detect fibrinogen-containing ICs. RA and control plasma were separated by liquid chromatography, and fractions then characterised by ELISA, immunoblotting and mass spectrometry. Immunohistochemical staining was performed on rheumatoid synovial tissue.

**Results:**

C1q-immunoassays demonstrated increased levels of IgG (p = 0.01) and IgM (p = 0.0002) ICs in plasma derived from RA patients possessing anti-cyclic citrullinated peptide (CCP+) autoantibodies as compared with healthy controls. About one-half of the anti-CCP+ RA possessed circulating ICs containing fibrinogen (p = 0.0004). Fractionation of whole RA plasma revealed citrullinated fibrinogen in the high molecular weight fractions that contained ICs. Positive correlations were observed between fibrinogen-containing ICs and anti-citrullinated fibrinogen autoantibodies, anti-CCP antibody, rheumatoid factor and certain clinical characteristics. Immunohistochemical staining demonstrated co-localisation of fibrinogen, immunoglobulin and complement component C3 in RA pannus tissue. Mass spectrometry analysis of immune complexes immunoprecipitated from RA pannus tissue lysates demonstrated the presence of citrullinated fibrinogen.

**Conclusion:**

Circulating ICs containing citrullinated fibrinogen are present in one-half of anti-CCP+ RA patients, and these ICs co-localise with C3 in the rheumatoid synovium suggesting that they contribute to synovitis in a subset of RA patients.

## Introduction

Rheumatoid arthritis (RA) is a chronic autoimmune synovitis affecting 0.6% of the world's population [1], yet the mechanisms underlying the initiation and progression of RA are still not completely understood. The presence of immune complexes (ICs) in the blood and synovial fluid of patients with RA is well described [2,3], and there is evidence they are involved in the activation of the complement cascade in RA synovial tissue [4]. However, apart from rheumatoid factor (RF) [5] and anti-collagen type II [6], the identity of the antigens involved in ICs in RA remains obscure.

Studies suggest critical roles for protein citrullination, B cells and autoantibodies in the pathogenesis of RA [7-10]. Citrullination is the post-translational conversion of arginine to citrulline, and in RA autoantibodies targeting cyclic citrullinated peptide (CCP) provides a sensitivity of approximately 70% and a specificity of 97% for the diagnosis of RA [7,11,12]. The citrullinated α- and β-chains of fibrin have been identified as potential targets of the autoantibody response in RA [13] and citrullinated fibrinogen is detected in RA synovial fluid [14].

Korganow and colleagues identified ICs involving glucose-6-phosphate isomerase (GPI) as mediating joint inflammation in their spontaneous K/BxN model [15]. These mice produce anti-GPI antibodies that form ICs that are deposited on articular surfaces and activate the alternative complement pathway to cause synovitis. Although studies suggest that GPI is not a specific autoantigen in RA [16], it is possible that the mechanisms involved in anti-GPI antibody arthritis and IC arthritis are relevant to a subset of human RA patients.

RA is characterised by excessive generation and breakdown of fibrinogen [17]. The citrullinated α- and β-chains of fibrin have also been identified as a potential target of the autoantibody response in RA [13,18] and citrullinated fibrinogen has been identified in synovial fluid derived from RA patients [14]. Autoantibodies against citrullinated fibrinogen have been described to provide diagnostic value in arthritis [18,19]. We previously generated synovial microarrays containing more than 500 proteins and peptides representing candidate autoantigens in RA, including protein and overlapping peptides representing native and citrullinated fibrinogen. Synovial microarray analysis demonstrated targeting of citrullinated fibrinogen in RA [20].

The methods described for the detection of ICs include chemical precipitation methods from as far back as the 1960s [21] and biological methods such as precipitation with Clq [22]. We adapted C1q capture immunoassays to utilise fibrinogen-specific secondary antibodies to identify fibrinogen-containing ICs, and applied these immunoassays to plasma samples derived from RA and control patients.

In the present study, we further investigated the targets of the autoantibody response and the antigens incorporated in ICs in RA. We demonstrated that one-half of anti-CCP+ RA patients possessed circulating (blood) ICs containing citrullinated fibrinogen, and that fibrinogen, immunoglobulin and complement component C3 co-localize in pannus tissue derived from RA patients. These data suggest that autoantibody targeting of citrullinated fibrinogen results in the formation of fibrinogen-containing ICs that characterise a subset of anti-CCP+ RA patients and may contribute to synovitis in RA.

## Materials and methods

### Human samples

All RA and control plasma and joint samples were obtained and studied with informed consent under Institutional Review Board approved protocols. The plasma samples used came from the Multiple Autoimmune Disease Genetics Consortium [23] and the Stanford Arthritis Center, collected in EDTA tubes (Table 1). The diagnosis of RA was made based on the American College of Rheumatology 1987 criteria [24].

**Table 1:** Source and description of samples used in the study

**Sample source**	**Disease**	**Number**	**Female, no. (%)**	**Age (range)**	**anti-CCP positive, no. (%)**	**RF positive, no. (%)**
Dr. P. Gregersen, plasma set 1	RA	30	28 (93)	72.6 (51 to 89)	20 (67)	24 (80)
	Healthy	10				

Dr. P. Gregersen, plasma set 2	IBD	20	13 (65)	46.4 (23 to 82)		
	JRA	20	16 (80)	37.3 (10 to 71)	6 (30)	6 (30)
	PsA	14	11 (79)	52.6 (23 to 75)		
	PS	20	10(50)	55.3(22 to 86)		
	RA	20	19(95)	59.0(35 to 89)		
	SLE	20	13 (65)	51.3 (29 to 67)		

CCP, cyclic-citrullinated peptides; RF, rheumatoid factor; RA, rheumatoid arthritis; IBD, inflammatory bowel disease; JRA, juvenile rheumatoid arthritis; PsA, psoriatic arthritis; PS, psoriasis; SLE, systemic lupus erythematosus.

### Mass spectrometry analysis

For in-gel digestion, protein spots were excised from the gel and treated with trypsin overnight at 37°C. The tryptic peptides were resolved by high-performance liquid chromatography (HPLC) using a Zorbax 300SB-C18 nanocolumn (Agilent Technologies, Palo Alto, CA, USA) packed with 3.5 μm particles (Agilent Technologies, Palo Alto, CA, USA) and eluted at 300 nL/minute with a 60 minute linear gradient from 0 to 95% acetonitrile containing 0.1% formic acid. Separated peptides were electrosprayed into an ion trap mass spectrometer (XCT Plus, Agilent Technologies, Palo Alto, CA, USA). For ICs immunoprecipitated from RA pannus tissue lysates, the precipitated complexes were directly digested with trypsin before mass spectrometry analysis. Proteins were identified based on raw MS/MS data compared with a SwissProt database using Mascot (Matrix Science, UK) with valid peptide hits.

### Detection of anti-citrullinated fibrinogen autoantibodies

Native fibrinogen (Calbiochem, San Diego, CA, USA) was citrullinated *in vitro *with a peptidylarginine deiminase derived from rabbit skeletal muscle (Sigma, St. Louis, MO, USA) using protocols previously described [25]. Anti-citrullinated fibrinogen autoantibodies were assayed as previously described [13,26,27]. Briefly, native fibrinogen or citrullinated fibrinogen was coated on ELISA plates (MaxiSorp; Nunc, Rochester, NY, USA) overnight at 4°C at a concentration of 20 μg/mL. Subsequent incubations and washes were performed at room temperature. The plates were blocked with 3% bovine serum albumin (BSA) in phosphate buffered saline with 0.05% Tween-20 (PBST) (Sigma, St. Louis, MO, USA) for one hour, washed and incubated with centrifuged plasma (diluted 50-fold) on a shaker for 1.5 hours. Anti-fibrinogen autoantibody was detected using horseradish peroxidase (HRP)-conjugated secondary reagents specific for human IgG (γchain) or IgM (μchain) specific antibodies diluted to 1:20,000.

### Quantitation of immune complexes

ELISA plates were coated with 20 μg/mL C1q (Sigma, St. Louis, MO, USA) in phosphate buffered saline (PBS) overnight at 4°C. Subsequent incubations and washes were performed at room temperature. The plates were blocked with 3% BSA in PBST for one hour. After washing, plasma from RA patients or healthy controls were diluted to 1:50 in PBST and incubated on a shaker for 1.5 hours. ICs were detected with HRP-conjugated rabbit antiserum specific for human IgG or IgM (Jackson Immunoresearch, West Grove, PA, USA).

### Quantitation of fibrinogen-containing immune complexes

ELISA plates coated with C1q were blocked with 3% BSA in PBST for one hour. After washing, plasma from RA patients or healthy controls was diluted to 1:10 and incubated on a shaker for 1.5 hours. Fibrinogen contained within the captured ICs was detected using a 1:4000 dilution of HRP-conjugated rabbit anti-human fibrinogen antiserum (Dako, Carpinteria, CA, USA).

### Anti-CCP and RF (IgM) ELISA

The anti-CCP (Euro Diagnostica, Malmö, Sweden) and RF ELISA kits (Alpha Diagnostic International, San Antonio, TX, USA) were used according to the manufacturers' protocol, except that plasma was used instead of serum. Anti-CCP and RF values of the samples were expressed as IU/mL.

### Fractionation of plasma samples

The plasma samples were filtered by a 0.45 μm cellulose acetate membrane in a Spin-x centrifuge tube filter (Corning, Corning, NY, USA) to remove cell debris and precipitates. A volume of 150 μL of the filtered plasma sample was injected into a fast protein liquid chromatography (FPLC) system (GE Healthcare Bio-Sciences, Piscataway, NJ, USA) equipped with a Superdex 200 10/300 gel filtration column (Amersham Biosciences, Piscataway, NJ, USA). A mixture of protein standard containing human fibrinogen, human albumin and IgG was run in parallel to further identify different peaks. All liquid chromatography runs were programmed at a flow rate of 0.4 mL/minute with PBS and fractions of 0.5 mL were collected. To measure total protein content of the fractions, 20 μL of each fraction was mixed with 100 μL of BCA buffer (Pierce Biotechnology, Rockford, IL, USA) and the mixture was incubated at 37°C for 30 minutes before the results were read at 562 nm on a spectraMAX190 instrument (Molecular Devices, Sunnyvale, CA, USA). To measure IgG and fibrinogen ICs, 50 μL of each fraction was applied to the C1q ELISA described above. To measure total IgG and fibrinogen content, the fractions were first diluted 10-fold with PBS. Then 1 μL of the dilutes was deposited onto a nitrocellulose membrane and left to dry overnight. After blocking with 5% milk in PBST, HRP-conjugated anti-human IgG or anti-human fibrinogen was applied to the membranes. Detection was carried out with SuperSignal West Pico Substrate (Pierce Biotechnology, Rockford, IL, USA). The densitometry of exposed film were measured with FluorChem imaging system (Alpha Innotech, San Leandro, CA, USA).

### Immunoblot

Plasma fractions were further separated with Precast Criterion Tris-HCl gels (4 to 20% linear gradient; Bio-Rad, Hercules, CA, USA), and separated proteins blotted onto nitrocellulose membranes. After blocking with 3% BSA in PBS, sera from RA patients or healthy controls were used to probe the membranes. Bound antibodies were detected with HRP-conjugated anti-human IgG (Jackson Immunoresearch, West Grove, PA, USA) using a SuperSignal kit (Pierce biotechnology, Rockford, IL, USA) and chemiluminescence was imaged with FluorChem imaging system (Alpha Innotech, San Leandro, CA, USA). Immunoblot with anti-modified citrulline was performed with an anti-citrulline detection kit (Upstate, Chicago, IL, USA) according to the manufacturer's instructions [13,27].

### Immunohistochemistry

Slides were deparaffinised and hydrated with water. Endogenous peroxidase was inhibited with 3% hydrogen peroxide, and non-specific staining blocked with DAKO Protein Block Serum-Free (Dako, Carpinteria, CA, USA). Staining for complement C3 was performed using a 1:2000 dilution of rabbit polyclonal antibodies against human complement C3 (Dako, Carpinteria, CA, USA). For fibrinogen and IgG staining, pretreatment of proteinase K (Dako, Carpinteria, CA, USA) was used before the primary antibody incubation. Slides positive for fibrinogen were immunohistochemically stained with a rabbit polyclonal antibody against human fibrinogen (Dako, Carpinteria, CA, USA), at room temperature at a dilution of 1:1600 for 30 minutes. After incubation with primary antibody, the tissue sections were sequentially incubated with Dako Envision+ Rabbit System Labeled Polymer HRP (Dako, Carpinteria, CA, USA) or biotinylated rabbit anti-goat antibodies (Vector, Burlingame, CA, USA) followed by streptavidin HRP (Dako, Carpinteria, CA, USA). Staining was developed with Liquid DAB+ (Dako, Carpinteria, CA, USA) and counterstained with haematoxylin and eosin.

### Statistics

All statistics were run using InStat™ software (GraphPad Software Inc., San Diego, CA, USA). For quantitation of ICs and autoantibodies to fibrinogen, unpaired *t*-tests with Welch correction were used.

## Results

### Identification of fibrinogen-containing circulating immunecomplexes in RA

**Figure 1:**
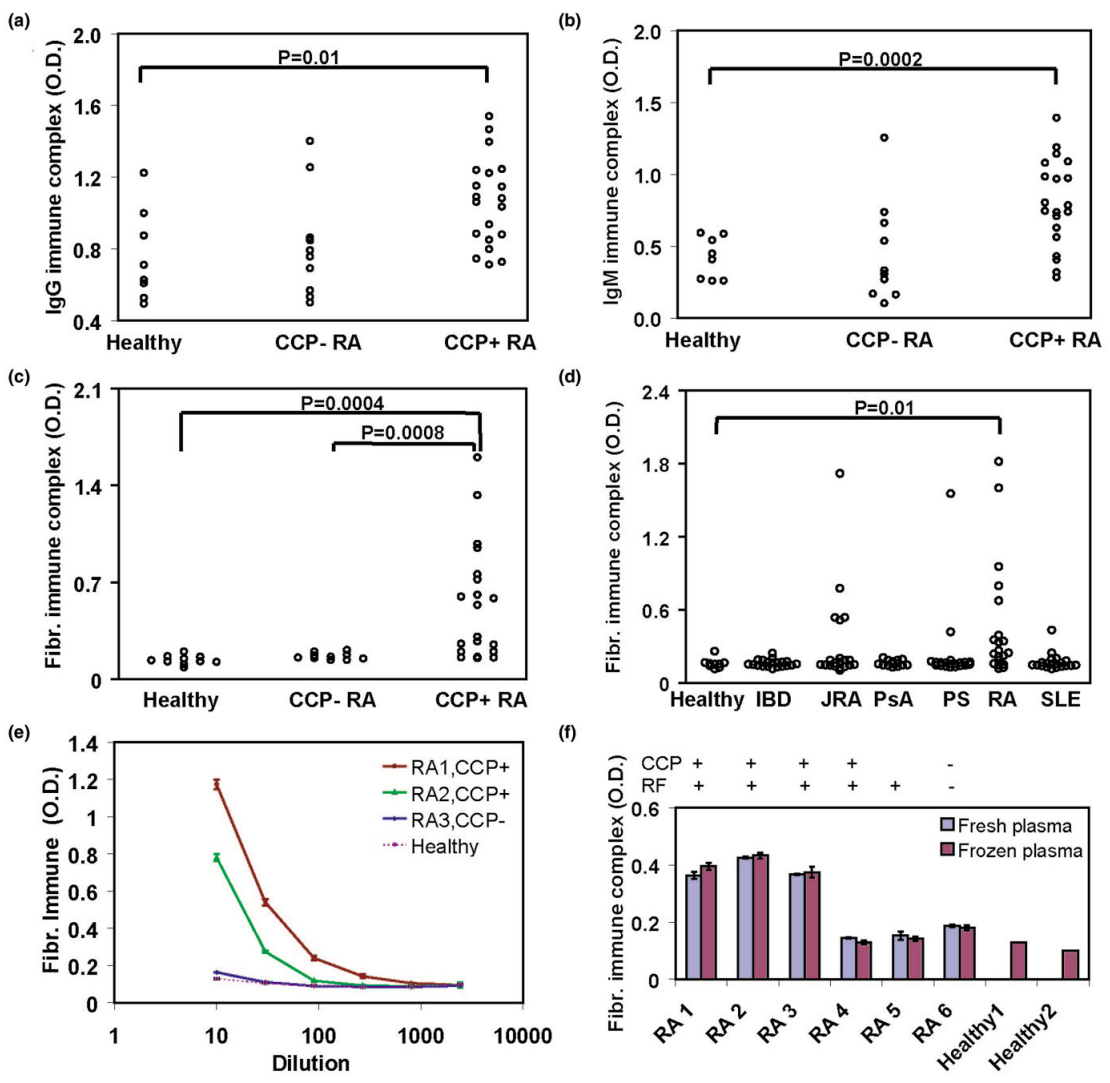
Fibrinogen-containing circulating immune complexes (ICs) in rheumatoid arthritis (RA). Circulating **(a) **IgG and **(b) **IgM ICs were detected in plasma derived from healthy individuals and anti-cyclic-citrullinated peptides (CCP) – and anti-CCP+ RA patients. ELISA plates were coated with C1q, incubated with 1:50 dilutions of plasma samples and horseradish peroxidase (HRP)-conjugated (a) anti-IgG or (b) anti-IgM secondary antibodies were used to detect the immunoglobulin isotypes contained in ICs. **(c, d) **Circulating fibrinogen-containing ICs were detected using HRP-conjugated fibrinogen-specific antisera as the secondary reagent. Statistical comparisons are based on an unpaired *t*-test with Welch correction. **(e) **Fibrinogen-containing ICs were detected with different dilutions of RA patient samples and healthy controls. Error bars represent the standard deviation of results from triplicate wells. **(f) **Fibrinogen-containing ICs were detected in fresh plasma and freeze-thawed plasma samples with no significant differences in values (data for anti-CCP status of RA5 is not available).

C1q binds aggregated immunoglobulin Fc regions and has been used to capture and quantitate ICs [28]. We used C1q-capture immunoassays and HRP-labelled secondary antibodies specific for human IgG and IgM to quantitate circulating ICs in plasma derived from anti-CCP+ RA, anti-CCP- RA and healthy control patients (Figures 1a,b). Elevated circulating IgG (p = 0.01; Figure 1a) and IgM (p = 0.0002; Figure 1b) ICs were observed in anti-CCP+ RA patients when compared with healthy controls. Most anti-CCP- RA patients did not possess circulating ICs (Figures 1a,b).

To determine if circulating ICs containing fibrinogen are present in RA, a fibrinogen-specific secondary antibody was used after C1q capture. One-half of anti-CCP+ RA patients possessed fibrinogen-containing ICs when compared with healthy controls (p = 0.0004) and anti-CCP- RA patients (p = 0.0008) (Figure 1c). Anti-CCP+ RA patients showed elevated titres of fibrinogen-containing ICs relative to anti-CCP- RA and healthy controls with low standard deviations (Figure 1e).

To further demonstrate that the observed fibrinogen-containing ICs did not result from non-specific binding of fibrinogen to immobilised C1q, we immobilised anti-C1q monoclonal antibodies to capture the C1q-bound ICs followed by detection with anti-fibrinogen antibodies. Similar results were obtained with anti-C1q monoclonal antibody capture as compared with C1q capture of ICs, and yielded a R^2 ^value of 0.9 in a linear regression analysis of the two assays (data not shown). Fibrinogen-containing ICs were also analysed from freshly collected plasma samples from both anti-CCP+ RA and anti-CCP- RA patients (within two hours of blood draw using EDTA plasma collection tubes). Compared with the same samples after a freeze-thaw cycle, no difference was detected (Figure 1f).

To demonstrate that fibrinogen-containing ICs were specifically detected in RA compared with other autoimmune diseases, these ICs were analysed from plasma samples collected in a panel of healthy (n = 10), inflammatory bowel disease (n = 20), juvenile RA (n = 20), psoriatic arthritis ([PsA] n = 14), psoriasis (n = 20), systemic lupus erythematosus (n = 20), and RA (n = 20) patients (Figure 1d). A subset of RA and a small subset of juvenile RA patients exhibited elevated circulating ICs containing fibrinogen, while patients with other autoimmune diseases did not (Figure 1d). The subset of juvenile RA patients possessing circulating ICs containing fibrinogen also possessed anti-citrullinated fibrinogen antibodies, RF and anti-CCP antibodies (Table 2). Chart reviews performed on this subset of juvenile RA patients revealed that they exhibited symmetrical polyarthritis (Table 2). These observations suggest that this subset of 'juvenile RA' patients in fact have adult RA, and is consistent with prior reports of 13% of juvenile RA patients exhibiting anti-CCP antibodies and clinical features consistent with adult RA [29].

**Table 2:** Clinical and laboratory characteristics of the juvenile rheumatoid arthritis (JRA) patients characterised

**Sample**	**Clinical features and rheumatoid factor status**	**Age** **Onset**	**Age** **History**	**Fibrinogen ICs (O.D.)**	**Anti-cit. fibrinogen IgG (O.D.)**	**anti-CCP^a^** **(IU/mL)**	**RF^b^** **(IU/mL)**
JRA 1	Polyarthritis, RF-	2	2	0.18	0.11	21.3	11.7
JRA 4	Polyarthritis, RF+	13		0.77	1.25	841.1	127.6
JRA 8	Polyarthritis, RF+	3	16	0.53	0.43	483.2	262.6
JRA 13	Polyarthritis, RF+	13	13	0.54	1.45	255.4	274.7
JRA 17	Systemic arthritis			0.15	0.10	24.8	24.9
JRA 22	Polyarthritis, RF-	15	15	0.13	0.09	22.7	27.1
JRA 27	Polyarthritis, RF-	1	2	0.10	0.08	20.3	7.6
JRA 31	Persistent oligoarthritis			0.16	0.13	21.5	7.3
JRA 32	Polyarthritis, RF-	5	5	0.12	0.37	26.7	6.3
JRA 41	Enthesitis-related arthritis	13	14	0.14	0.08	20.8	13.1
JRA 42	Systemic arthritis	12	12	0.17	0.08	21.8	7.1
JRA 44	Polyarthritis, RF-	9	9	0.19	0.09	20.9	10.1
JRA 49	Polyarthritis, RF+	9		1.72	0.74	1275.4	295.2
JRA 51	Systemic arthritis	5	5	0.14	0.09	21.4	7.6
JRA 71	Polyarthritis, RF+		10	0.19	0.21	360.9	186.1
JRA 79	Polyarthritis, RF-	4	4	0.20	0.10	22.1	11.7
JRA 88	Polyarthritis, RF+	11	11	0.51	0.35	287.0	236.4
JRA 106	Extended oligoarthrits	1	3	0.15	0.12	22.0	10.0
JRA 110	Persistent oligoarthritis			0.15	0.10	24.9	14.6
JRA 112	Enthesitis related arthritis			0.14	0.12	22.5	15.9

^a,b^Measured with commercial kits. JRA, juvenile rheumatoid arthritis; IC, immune complex; CCP, cyclic-citrullinated peptides; RF, rheumatoid factor; O.D., optical density

### Liquid chromatographic separation demonstrates co-fractionation of citrullinated fibrinogen with immune complexes

**Figure 2:**
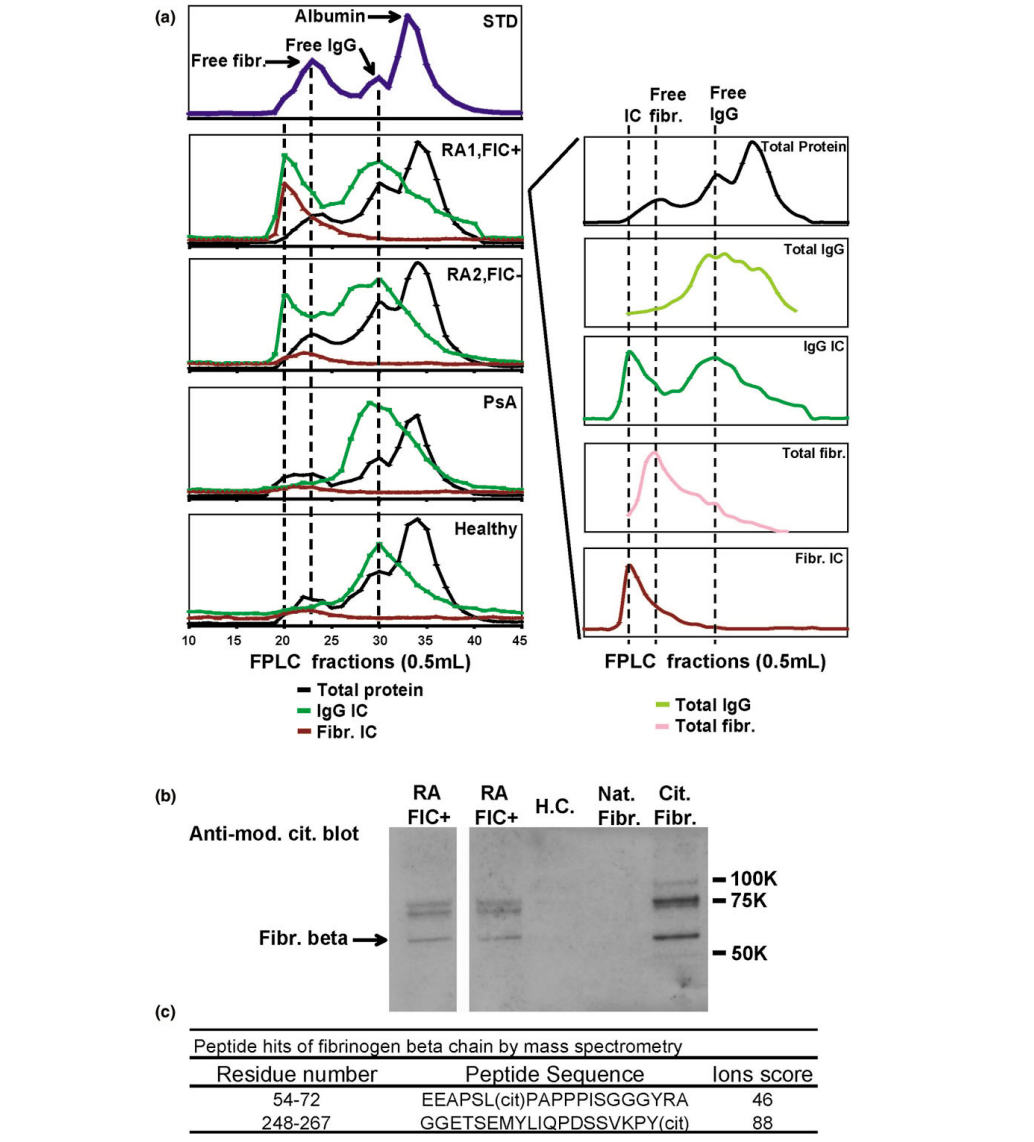
Citrullinated fibrinogen-containing immune complexes (ICs) are separated from rheumatoid arthritis (RA) plasma. **(a) **Liquid chromatographic separation of RA plasma. Fast protein liquid chromatography (FPLC) was used to fractionate plasma derived from RA and control patients. Forty-five fractions were collected from each plasma sample, and individual fractions were analysed for total protein, fibrinogen, IgG, IgG ICs and fibrinogen ICs (FIC), and relative levels of each of these components are plotted. Plasma samples from two RA patients (RA1 and RA2), a psoriatic arthritis (PsA) patients and a healthy control were characterised. The right panel presents individual traces from patient RA1, with the dashed lines indicating the fractions containing the peak levels of ICs, free fibrinogen and free Ig. **(b) **Citrullinated fibrinogen was identified by anti-modified citrulline blot. **(c) **In-gel trypsin digestion of the bands followed by mass spectrometry revealed two citrullinated peptides derived from beta chain of human fibrinogen.

To demonstrate that the fibrinogen-containing ICs in plasma are physically distinct from free fibrinogen and free immunoglobulin, we used size exclusion chromatography as previously described [16]. Size exclusion chromatography was applied to fractionate plasma derived from an RA patient with fibrinogen-containing circulating ICs, an RA patient with circulating ICs but not fibrinogen-containing circulating ICs, a PsA patient and a healthy control (Figure 2a). Forty-five fractions were generated of each patient's plasma and each fraction was assayed for ICs, fibrinogen-containing ICs, total immunoglobulin, total fibrinogen and total protein.

ELISA analysis of the fractions containing IgG ICs showed two peaks in the elution profile of both RA samples, but not in the corresponding fractions from the PsA and healthy control samples (Figure 2a, green line). The first peak (RA1 and RA2, green line) corresponded to the first three fractions after the void volume, which had a molecular mass of 300 kD or higher, and corresponded to the fractions in which ICs eluted. The second peak (RA1 and RA2, green line) corresponded to free IgG as compared with a chromatography run of standards (STD, blue line). ELISA analysis of fibrinogen ICs on RA1 (which possessed fibrinogen ICs) showed a single peak (Figure 2a, RA1, red line) that was eluted in the same fractions at the IgG IC peak (Figure 2a, RA1 and RA2, first peak of green line). Similar analysis on RA2 did not show a co-eluted peak (RA2, red line).

To further determine that fibrinogen detected from IC fractions was not a contamination from free fibrinogen in blood, free fibrinogen from each RA1 fraction was quantitated by dot assay (Figure 2a, right panel, pink line). The peak of free fibrinogen was well separated from the peak of fibrinogen IC (Figure 2a, right panel, red line), as shown by the first two dotted lines. PsA and healthy control patients did not possess circulating ICs (PsA and healthy, green line). It is possible that following the collection of the plasma fractions, that the free IgG fractions that contained high levels of IgG developed some IgG aggregates that were then detected by the IgG IC assay. These results demonstrate that the fibrinogen-containing circulating ICs observed co-elute with the IgG ICs, and that the fractions containing fibrinogen-ICs are distinct from those containing free fibrinogen and free immunoglobulin.

To determine if the fibrinogen present in circulating ICs is citrullinated, the FPLC fractions that contained fibrinogen ICs were separated by sodium dodecyl sulfate polyacrylamide gel electrophoresis (SDS-PAGE) and immunoblotted with anti-modified citrulline antibody (Figure 2b). Citrullinated polypeptides that co-migrated with fibrinogen polypeptides were detected only in the fractions derived from RA patients but not in the corresponding fractions isolated from controls. The band, indicated as fibrinogen beta chain, was further analysed by mass spectrometry. Two distinct citrullinated peptides from the fibrinogen beta chain were identified (Figure 2c).

### Laboratory and clinical features associated with fibrinogen-containing circulating immune complexes

**Figure 3:**
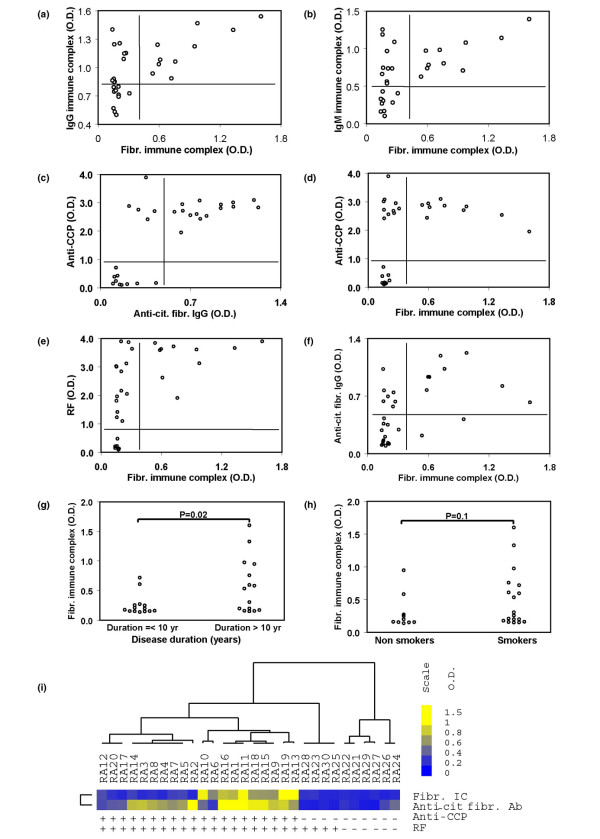
Fibrinogen-containing circulating immune complexes (ICs) are associated with anti-cyclic-citrullinated peptides (CCP) antibodies, rheumatoid factor (RF) and a disease duration of more than 10 years. Scatter plots are presented for the association of **(a, b) **fibrinogen ICs with IgG and IgM ICs; **(c) **anti-citrullinated fibrinogen antibodies with anti-CCP antibodies; **(d) **fibrinogen ICs with anti-CCP antibodies; **(e) **RF; and **(f) **anti-citrullinated fibrinogen. Lines were drawn to mark the negative and positive measurements of each species. **(g, h) **Levels of fibrinogen ICs are also plotted in RA patients with (g) more than 10 years disease duration and (h) smoking history. **(i) **Unsupervised hierarctical clustering [54] of 30 RA patients and levels of fibrinogen-circulating ICs, anti-citrullinated fibrinogen antibodies, RF and anti-CCP are presented as a heatmap. Tree dendrograms represent the statistical relatedness between patients.

We observed positive correlations between fibrinogen-containing ICs with IgG and IgM ICs, anti-citrullinated fibrinogen antibodies, anti-CCP antibodies, RF and certain clinical characteristics (Figures 3a–h). Of anti-CCP+ RA patients, three-quarters possess anti-citrullinated fibrinogen antibodies (Figures 3c, i) and one-half possess fibrinogen-containing circulating ICs (Figures 3d, i). All patients with fibrinogen-containing circulating ICs possess RF, while more than one-half of RF+ patients did not possess fibrinogen-containing ICs (Figures 3e, i). Interestingly, fibrinogen-containing ICs were not detected in a subset of the RA patients who possessed high IgG and IgM plasma ICs, suggesting that circulating ICs containing other antigens are present in this subset of RA patients (Figures 3a,b). In RA patients, the presence of circulating ICs containing fibrinogen was associated with a disease duration of more than 10 years (p = 0.02; Figure 3g), and there were trends towards associations with smoking (p = 0.1; Figure 3h).

Unsupervised hierarchical clustering of 30 RA patients based on their anti-CCP antibody, RF, anti-citrullinated fibrinogen antibody and fibrinogen-containing circulating IC levels demonstrates statistical groupings (Figure 3i). The anti-CCP+ RF+ patients cluster together, and more than one-half of these patients possess anti-citrullinated fibrinogen autoantibodies and circulating ICs containing fibrinogen.

### Immunohistochemistry demonstrates co-staining of fibrinogen, complement component C3, and immunoglobulin in pannus tissue derived from RA patients

**Figure 4:**
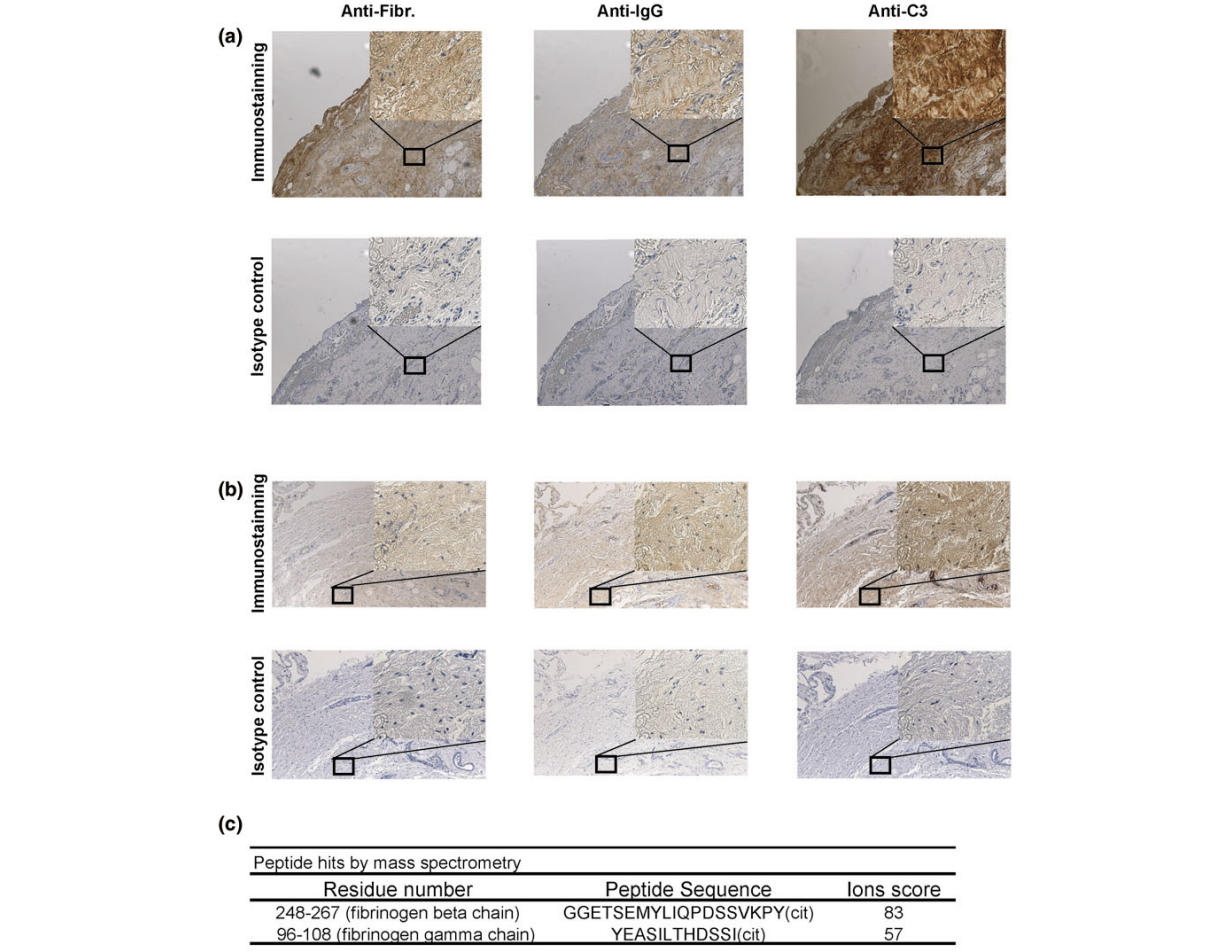
Synovial tissue immune complexes (ICs) contain citrullinated fibrinogen. **(a, b) **immunohistochemistry demonstrates co-localisation of fibrinogen, complement component C3 and immunoglobulins in rheumatoid arthritis (RA) pannus tissue. Representative staining of synovium derived from two separate cyclic-citrullinated peptides (CCP) + rheumatoid factor (RF) + RA patients are shown in (a) and (b). Immunohistochemistry was performed on articular cartilage samples derived from RA patients. Samples were fixed, paraffin-embedded and sections stained with antisera specific for complement component C3, fibrinogen and IgG, as well as with matched pre-immune sera. Horseradish peroxidase (HRP)-conjugated secondary antibodies were utilised to detect primary antibody reactivity. These stainings demonstrate co-localisation of complement component C3, fibrinogen and immunoglobulin staining at the surface of the articular cartilage sections. **(c) **Mass spectrometry analysis of ICs immunoprecipitated from RA synovial tissue demonstrates the presence of citrullinated fibrinogen peptides.

To further investigate the role of fibrinogen-containing ICs in RA, we performed immunohistochemistry on remnant pannus tissue derived from two anti-CCP+ RF+ RA patients. Pannus tissue was obtained from RA patients at the time of knee arthroplasty, fixed and sectioned, then consecutive sections were stained with antibodies specific for complement component C3, fibrinogen and immunoglobulin. Representative results are presented from the analysis of consecutive sections of pannus derived from two independent patients. Immunohistochemical staining demonstrates co-localisation of the complement component C3, fibrinogen, and IgG in both RA patients (Figures 4a,b).

RA synovial tissue was minced and the protein contents extracted with tissue protein extraction buffer. Lysates were immunoprecipitated with protein-G-sepharose to capture ICs present in the rheumatoid synovial tissue. These ICs were eluted from the protein-G beads, trypsinised and the trypsin digests directly analysed by mass spectroscopy to demonstrate the presence of citrullinated fibrinogen in ICs isolated from RA pannus tissue (Figure 4c). These data suggest that citrullinated-fibrinogen containing ICs either deposit or form in synovial tissue in RA. The co-localisation of citrullinated fibrinogen-containing ICs with complement component C3 in RA pannus further suggests that they could activate the complement cascade to cause synovitis in RA.

## Discussion

The presence of ICs in the blood and inflamed joints of patients with RA was described decades ago [30], and several recent findings have resulted in a resurgence of interest in the role of autoantibodies and B cells in RA. These findings include the facts that: anti-citrullinated protein autoantibodies can predate the development of clinical arthritis and provide a sensitivity of approximately 70% and a specificity of 97% for the diagnosis of RA [7,11,12,31]; anti-CD20-mediated B cell depletion provides efficacy in treating RA [9]; and the K/BxN mouse model develops spontaneous arthritis mediated by antibodies targeting the ubiquitous glycolytic enzyme GPI [32]. Although ICs have been isolated from RA patient plasma by means of polyethylene glycol precipitation [33] and C1q affinity columns [22], the identity of the antigens incorporated in these ICs is not well defined. In the present study we characterise circulating and synovial tissue ICs, and demonstrate the presence of circulating ICs containing fibrinogen in one-half of anti-CCP+ RA patients (Figure 1). We used immunoblotting and mass spectroscopy to demonstrate that the fibrinogen contained in these circulating ICs is citrullinated (Figures 2b,c), and that ICs isolated from RA pannus tissue also contain citrullinated fibrinogen (Figure 4c). Finally, we demonstrate co-localisation of complement component C3, fibrinogen and immunoglobulin in RA pannus tissue (Figures 4a,b), suggesting that these complexes contribute to synovitis in RA.

Although it is difficult to completely exclude the possibility that the ICs detected are formed *in vivo *rather than *ex vivo*, Figure 1f provides reassurance that freezing and freeze-thaw are not responsible for the observed ICs. Further, fibrinogen-containing ICs were not observed in plasma derived from patients with a variety of other inflammatory arthritidies for which the plasma was collected and stored alongside the anti-CCP+ RA plasma in which fibrinogen ICs were demonstrated (Figures 1c,d). Although complement containing ICs usually bind to erythrocytes and are transported to the liver for clearance, in plasma derived from anti-CCP+ RA patients we found circulating C1q-bound ICs that contain fibrinogen. Further, fibrinogen ICs were also detected by anti-C1q monoclonal antibody capture and results were concordant with our results from C1q capture of ICs (comparison of results yielded a R^2 ^value of 0.9 in linear regression; data not shown).

There is growing evidence that fibrin could be an important autoantigen in RA [13,18]. Consistent with previous findings [34], autoantibody reactivity is only observed against citrullinated fibrinogen, and not against its native form (data not shown). Although the anti-citrullinated fibrinogen antibodies observed in RA do not result in overt clinical haematological manifestations, RA is characterised by extravascular coagulation and the accumulation of fibrin in the arthritic joint [17,35]. It has been hypothesised that a local imbalance between coagulation and fibrinolysis contributes to pathogenesis, and it is possible that autoantibodies targeting citrullinated fibrinogen could contribute to this imbalance by altering the structural and/or functional properties of fibrinogen and/or fibrin.

Fibrin is one of the classical citrulline-modified proteins [36], and the presence of citrullinated fibrinogen and/or fibrin has been demonstrated in the rheumatoid joint [14,37]. Nevertheless, citrullinated fibrinogen is generated in inflamed synovia arising from a variety of inflammatory conditions [37]. Our observation that ICs containing citrullinated fibrinogen are present in the plasma of anti-CCP+ RA patients, but not in plasma derived from anti-CCP- RA, anti-CCP-juvenile RA and PsA patients (Figures 1 and 2), suggests a potential role for citrullinated fibrinogen-containing circulating ICs in RA. Our mass spectrometry analysis of the fibrinogen contained in circulating ICs derived from anti-CCP+ RA patients demonstrated a few citrullinated peptides from the α-chain of fibrinogen, but these peptides were not included in Figure 2c because of low Mascot scores. Our results are consistent with several previous publications that describe citrullinated epitopes derived from the beta, but not the alpha, chain of fibrinogen [38,39]. Although trypsin has been described to be incapable of cleaving C-terminal to citrulline residues [40], two of the three citrullinated peptides identified contain a citrulline at the C-terminus (Figures 2c and 4c). Using mass spectrometry analysis, we have detected multiple citrullinated peptides with C-terminal citrullines (as well as non-C terminal citrullines) in tryspin digests of multiple different citrullinated proteins in several experiments. In addition, citrullinated peptides with C-terminal citrullines were also observed from multiple citrullinated proteins that were sent to and analysed by an independent mass spectrometry core facility. The explanation for this observation remains unclear, and it is possible that our results are due to altered trypsin cleavage, which is polypeptide sequence and/or trypsin reaction condition dependent. The highly significant Mascot scores of our reported citrullinated peptides (Figures 2c and 4c) support the validity of our results.

The excessive formation of fibrin in the rheumatoid joint in combination with its citrullination and structural properties that include repetitive antigenic motifs, could result in activation of B cells specific for citrullinated fibrinogen via cross-linking of surface immunoglobulin receptors. Citrullination of collagen was demonstrated to increase its immunogenicity and arthritogenicity in a rat arthritis model [41]. Recently, immunisation with citrullinated fibrinogen was described to induce arthritis in human leucocyte antigen (HLA) DR4-IE expressing transgenic mice, demonstrating the arthritogenic potential of citrullinated fibrinogen in mice expression RA-associated major histocompatibility complex (MHC) class II molecules [42].

Fibrin and/or fibrinogen plays an important role in a variety of inflammatory and immunological processes. Multiple cells, including neutrophils and macrophages, express integrins and other receptors that bind fibrin and/or fibrinogen [43]. Fibrinogen has also been hypothesised to serve as a structural scaffold for the formation and growth of pannus [44]. Fibrinogen is chemotactic for endothelial cells that are involved in angiogenesis [45], which is integral to the formation of pannus. Fibrin deposits in RA synovial tissue are hypothesised to activate synovial fibroblast proliferation and cytokine release, as well as other inflammatory cell responses [17]. Fibrinogen has been shown to stimulate macrophage chemokine secretion through TLR-4 [46]. Further, it was recently demonstrated that RA-specific autoantibodies complexed to citrullinated fibrinogen stimulate macrophages to produce TNF via engagement of FcγRIIa [47].

Cantaert and colleagues suggested that the expression of citrullinated proteins is essential but not sufficient for the development of RA, and that generation of well-defined citrullinated epitopes is likely to play a critical role [48]. In this context, our results might suggest that the development of autoantibodies targeting citrullinated epitopes specific to fibrinogen might play an important role in the pathogenesis of RA. In further support of a potential pathogenic role for citrullinated fibrinogen in RA, it was recently demonstrated that citrullinated fibrinogen bound by autoantibodies present in RA patient sera stimulate macrophage through FcγRIIa to secrete TNF [47].

It was unexpected to observe autoantibodies targeting citrullinated fibrinogen along with fibrinogen-containing circulating ICs in a subset of juvenile RA patients (Figure 1 and Table 2). However, late-onset polyarticular juvenile RA is associated with RF-positivity in about 5% of patients, and has been considered to be identical to adult RA. Further, a recent report described 13% of polyarticular-onset juvenile RA patients exhibiting anti-CCP antibodies [29]. Following the observation of anti-citrullinated fibrinogen autoatibodies and fibrinogen-containing circulating ICs in a subset of juvenileRA patients (Table 2), we performed chart reviews with anti-CCP and RF ELISA tests on these plasma samples. Of the six juvenile RA patients exhibiting elevated levels of anti-citrullinated fibrinogen antibodies, all exhibited a symmetrical polyarthritis and possessed RF antibodies. All but one of the six anti-CCP+ and RF+ juvenile RA patients possessed high levels of fibrinogen ICs. The age of disease onset of the anti-CCP+ and RF+ juvenile RA patients were 13, 11, 3, 13 and 9 years, and these patients were relatively older than the other juvenile RA patients included in this cohort. Interestingly, five out of six anti-CCP+ and RF+ juvenile RA patients possessed fibrinogen-containing ICs, compared with only 50% of anti-CCP+ and RF+ adult RA patients. This observation suggests that anti-fibrinogen autoimmunity and fibrinogen-containing ICs play a significant role in this subset of juvenile RA patients. Characterisation of larger cohorts of juvenile RA patients will be necessary to validate and further investigate this observation.

Immunohistochemical analysis demonstrated co-localisation of the staining for fibrinogen, complement component C3 and immunoglobulin in serial sections derived from RA pannus tissue (Figure 4). These results suggest that fibrinogen-containing ICs deposit on or form in synovial lining tissue, and activate the complement cascade to cause inflammatory arthritis. In the K/BxN model, arthritis is mediated by anti-GPI antibodies and was demonstrated to depend on FcRγ and components of the alternative complement pathway [49]. It has been speculated that accumulation of ICs involving GPI may activate the alternative complement pathway to cause inflammatory arthritis [16]. We hypothesise that autoantibodies targeting citrullinated fibrinogen could result in IC-mediated arthritis based on mechanisms analogous to those observed in the K/BxN model [16] and via macrophage FcγRIIa-mediated TNF production [47].

Anti-citrullinated fibrinogen autoantibodies were detected in three-quarters of anti-CCP+ RA patients (data not shown) while fibrinogen containing ICs were found in one-half (Figures 1 and 3). These observations are consistent with RA being a clinically and molecularly heterogeneous disease, as evidenced by differential expression of anti-citrulline antibodies [7,11], variable responsiveness to anti-tumor necrosis factor (TNF) therapy [50] and heterogeneity in the genetic background of patients which includes polymorphisms in the MHC (major histocompatibility complex), *TRAF1-C5 *(encoding tumor necrosis factor receptor-associated factor 1 and complement component 5) [51], *STAT4 *(encoding signal transducer and activator of transcription 4) [51,52] and *PTPN22 *(encoding protein tyrosine phosphatase, non-receptor type 22) [53] genes. CCP is derived from filaggrin, a protein expressed by keratinocytes in the epidermis, and it is likely that autoantibody reactivity against the CCPs derived from filaggrin represents molecular cross reactivity. Our findings suggest that the development of citrullinated fibrinogen-containing ICs in RA synovial tissue activates the complement cascade and contributes to synovitis in RA.

## Conclusion

In summary, the data presented herein suggest that autoimmunity targeting citrullinated fibrinogen and the development of fibrinogen-containing ICs could contribute to synovitis in approximately one-half of anti-CCP+ RA patients. These results expand the possibility for the development of novel diagnostics as well as for the development of specific therapies for this subset of RA patients.

## Abbreviations

BSA = bovine serum albumin; CCP = cyclic-citrullinated peptides; FPLC = fast protein liquid chromatography; GPI = glucose-6-phosphate isomerase; HLA = human leucocyte antigen; HRP = horseradish peroxidase; IBD = inflammatory bowel disease; IC = immune complex; JRA = juvenile rheumatoid arthritis; MHC = major histocompatibility complex; PBS = phosphate buffered saline; PBST = phosphate buffered saline with 0.05% Tween-20; PS = psoriasis; PsA = psoriatic arthritis; RA = rheumatoid arthritis; RF = rheumatoid factor; SLE = systemic lupus erythematosus.

## Competing interests

The authors declare that they have no competing interests.

## Authors' contributions

X.Z. and W.H.R. conceived the studies, carried out the experiments, analyzed the data, and wrote the manuscript. N.L.O. and O.S. helped perform the mass spectrometry experiments. F.M.B., A.T.L. and P.K.G. provided human samples and clinical data, and contributed to interpretation of the data. P.P.H. and B.H.T. contributed to data analysis.

## References

[B1] Firestein GS (2003). Evolving concepts of rheumatoid arthritis. Nature.

[B2] Zubler RH, Nydegger U, Perrin LH, Fehr K, McCormick J, Lambert PH, Miescher PA (1976). Circulating and intra-articular immune complexes in patients with rheumatoid arthritis. Correlation of 125I-Clq binding activity with clinical and biological features of the disease. J Clin Invest.

[B3] Antes U, Heinz HP, Schultz D, Brackertz D, Loos M (1991). C1q-bearing immune complexes detected by a monoclonal antibody to human C1q in rheumatoid arthritis sera and synovial fluids. Rheumatol Int.

[B4] Low JM, Moore TL (2005). A role for the complement system in rheumatoid arthritis. Curr Pharm Des.

[B5] Newkirk MM, Fournier MJ, Shiroky J (1995). Rheumatoid factor avidity in patients with rheumatoid arthritis: identification of pathogenic RFs which correlate with disease parameters and with the gal(0) glycoform of IgG. J Clin Immunol.

[B6] Steffen C, Ludwig H, Knapp W, Thumb N, Eberl R, Frank O, Freilinger H (1975). Collagen antibodies and collagen-anticollagen immune complexes in rheumatoid arthritis. Z Rheumatol.

[B7] Schellekens GA, de Jong BA, Hoogen FH van den, Putte LB van de, van Venrooij WJ (1998). Citrulline is an essential constituent of antigenic determinants recognized by rheumatoid arthritis-specific autoantibodies. J Clin Invest.

[B8] Kuhn KA, Kulik L, Tomooka B, Braschler KJ, Arend WP, Robinson WH, Holers VM (2006). Antibodies against citrullinated proteins enhance tissue injury in experimental autoimmune arthritis. J Clin Invest.

[B9] Edwards JC, Szczepanski L, Szechinski J, Filipowicz-Sosnowska A, Emery P, Close DR, Stevens RM, Shaw T (2004). Efficacy of B-cell-targeted therapy with rituximab in patients with rheumatoid arthritis. N Engl J Med.

[B10] Agrawal S, Misra R, Aggarwal A (2007). Autoantibodies in rheumatoid arthritis: association with severity of disease in established RA. Clin Rheumatol.

[B11] Schellekens GA, Visser H, de Jong BA, Hoogen FH van den, Hazes JM, Breedveld FC, van Venrooij WJ (2000). The diagnostic properties of rheumatoid arthritis antibodies recognizing a cyclic citrullinated peptide. Arthritis Rheum.

[B12] Mimori T (2005). Clinical significance of anti-CCP antibodies in rheumatoid arthritis. Intern Med.

[B13] Masson-Bessiere C, Sebbag M, Girbal-Neuhauser E, Nogueira L, Vincent C, Senshu T, Serre G (2001). The major synovial targets of the rheumatoid arthritis-specific antifilaggrin autoantibodies are deiminated forms of the alpha- and beta-chains of fibrin. J Immunol.

[B14] Takizawa Y, Suzuki A, Sawada T, Ohsaka M, Inoue T, Yamada R, Yamamoto K (2006). Citrullinated fibrinogen detected as a soluble citrullinated autoantigen in rheumatoid arthritis synovial fluids. Ann Rheum Dis.

[B15] Korganow AS, Ji H, Mangialaio S, Duchatelle V, Pelanda R, Martin T, Degott C, Kikutani H, Rajewsky K, Pasquali JL, Benoist C, Mathis D (1999). From systemic T cell self-reactivity to organ-specific autoimmune disease via immunoglobulins. Immunity.

[B16] Matsumoto I, Maccioni M, Lee DM, Maurice M, Simmons B, Brenner M, Mathis D, Benoist C (2002). How antibodies to a ubiquitous cytoplasmic enzyme may provoke joint-specific autoimmune disease. Nat Immunol.

[B17] Sanchez-Pernaute O, Largo R, Calvo E, Alvarez-Soria MA, Egido J, Herrero-Beaumont G (2003). A fibrin based model for rheumatoid synovitis. Ann Rheum Dis.

[B18] Cruyssen B Vander, Cantaert T, Nogueira L, Clavel C, De Rycke L, Dendoven A, Sebag M, Deforce D, Vincent C, Elewaut D, Serre G, De Keyser F (2006). Diagnostic value of anti-human citrullinated fibrinogen ELISA and comparison with four other anti-citrullinated protein assays. Arthritis Res Ther.

[B19] Nielen MM, Horst AR van der, van Schaardenburg D, Horst-Bruinsma IE van der, Stadt RJ van de, Aarden L, Dijkmans BA, Hamann D (2005). Antibodies to citrullinated human fibrinogen (ACF) have diagnostic and prognostic value in early arthritis. Ann Rheum Dis.

[B20] Hueber W, Kidd BA, Tomooka BH, Lee BJ, Bruce B, Fries JF, Sønderstrup G, Monach P, Drijfhout JW, van Venrooij WJ, Utz PJ, Genovese MC, Robinson WH (2005). Antigen microarray profiling of autoantibodies in rheumatoid arthritis. Arthritis Rheum.

[B21] Kunkel HG, Muller-Eberhard HJ, Fudenberg HH, Tomasi TB (1961). Gamma globulin complexes in rheumatoid arthritis and certain other conditions. J Clin Invest.

[B22] Khalkhali-Ellis Z, Bulla GA, Schlesinger LS, Kirschmann DA, Moore TL, Hendrix MJ (1999). C1q-containing immune complexes purified from sera of juvenile rheumatoid arthritis patients mediate IL-8 production by human synoviocytes: role of C1q receptors. J Immunol.

[B23] Criswell LA, Pfeiffer KA, Lum RF, Gonzales B, Novitzke J, Kern M, Moser KL, Begovich AB, Carlton VE, Li W, Lee AT, Ortmann W, Behrens TW, Gregersen PK (2005). Analysis of families in the multiple autoimmune disease genetics consortium (MADGC) collection: the PTPN22 620W allele associates with multiple autoimmune phenotypes. Am J Hum Genet.

[B24] Arnett FC, Edworthy SM, Bloch DA, McShane DJ, Fries JF, Cooper NS, Healey LA, Kaplan SR, Liang MH, Luthra HS, Medsger TA, Mitchell DM, Neustadt DH, Pinals RS, Schaller JG, Sharp JT, Wilder RL, Hunder GG (1988). The American Rheumatism Association 1987 revised criteria for the classification of rheumatoid arthritis. Arthritis Rheum.

[B25] Vossenaar ER, Despres N, Lapointe E, Heijden A van der, Lora M, Senshu T, van Venrooij WJ, Menard HA (2004). Rheumatoid arthritis specific anti-Sa antibodies target citrullinated vimentin. Arthritis Res Ther.

[B26] Chapuy-Regaud S, Nogueira L, Clavel C, Sebbag M, Vincent C, Serre G (2005). IgG subclass distribution of the rheumatoid arthritis-specific autoantibodies to citrullinated fibrin. Clin Exp Immunol.

[B27] Senshu T, Akiyama K, Kan S, Asaga H, Ishigami A, Manabe M (1995). Detection of deiminated proteins in rat skin: probing with a monospecific antibody after modification of citrulline residues. J Invest Dermatol.

[B28] Agnello V, Winchester RJ, Kunkel HG (1970). Precipitin reactions of the C1q component of complement with aggregated gamma-globulin and immune complexes in gel diffusion. Immunology.

[B29] Ferucci ED, Majka DS, Parrish LA, Moroldo MB, Ryan M, Passo M, Thompson SD, Deane KD, Rewers M, Arend WP, Glass DN, Norris JM, Holers VM (2005). Antibodies against cyclic citrullinated peptide are associated with HLA-DR4 in simplex and multiplex polyarticular-onset juvenile rheumatoid arthritis. Arthritis Rheum.

[B30] Zvaifler NJ (1973). The immunopathology of joint inflammation in rheumatoid arthritis. Adv Immunol.

[B31] Nielen MM, van Schaardenburg D, Reesink HW, Stadt RJ van de, Horst-Bruinsma IE van der, de Koning MH, Habibuw MR, Vandenbroucke JP, Dijkmans BA (2004). Specific autoantibodies precede the symptoms of rheumatoid arthritis: a study of serial measurements in blood donors. Arthritis Rheum.

[B32] Matsumoto I, Staub A, Benoist C, Mathis D (1999). Arthritis provoked by linked T and B cell recognition of a glycolytic enzyme. Science.

[B33] Ferraccioli G, Karsh J, Osterland CK (1983). Immunochemical analyses of components of immune complexes in the sera of patients with autoimmune diseases. J Rheumatol.

[B34] Hill JA, Al-Bishri J, Gladman DD, Cairns E, Bell DA (2006). Serum autoantibodies that bind citrullinated fibrinogen are frequently found in patients with rheumatoid arthritis. J Rheumatol.

[B35] Busso N, Hamilton JA (2002). Extravascular coagulation and the plasminogen activator/plasmin system in rheumatoid arthritis. Arthritis Rheum.

[B36] Vossenaar ER, Zendman AJ, van Venrooij WJ, Pruijn GJ (2003). PAD, a growing family of citrullinating enzymes: genes, features and involvement in disease. Bioessays.

[B37] Chapuy-Regaud S, Sebbag M, Baeten D, Clavel C, Foulquier C, De Keyser F, Serre G (2005). Fibrin deimination in synovial tissue is not specific for rheumatoid arthritis but commonly occurs during synovitides. J Immunol.

[B38] Tilleman K, Van Steendam K, Cantaert T, De Keyser F, Elewaut D, Deforce D (2008). Synovial detection and autoantibody reactivity of processed citrullinated isoforms of vimentin in inflammatory arthritides. Rheumatology (Oxford).

[B39] Matsuo K, Xiang Y, Nakamura H, Masuko K, Yudoh K, Noyori K, Nishioka K, Saito T, Kato T (2006). Identification of novel citrullinated autoantigens of synovium in rheumatoid arthritis using a proteomic approach. Arthritis Res Ther.

[B40] Kurokawa T, Hara S, Takahara H, Sugawara K, Ikenaka T (1987). Conversion of peanut trypsin-chymotrypsin inhibitor B-III to a chymotrypsin inhibitor by deimination of the P1 arginine residues in two reactive sites. J Biochem.

[B41] Lundberg K, Nijenhuis S, Vossenaar ER, Palmblad K, van Venrooij WJ, Klareskog L, Zendman AJ, Harris HE (2005). Citrullinated proteins have increased immunogenicity and arthritogenicity and their presence in arthritic joints correlates with disease severity. Arthritis Res Ther.

[B42] Hill JA, Bell DA, Brintnell W, Yue D, Wehrli B, Jevnikar AM, Lee DM, Hueber W, Robinson WH, Cairns E (2008). Arthritis induced by posttranslationally modified (citrullinated) fibrinogen in DR4-IE transgenic mice. J Exp Med.

[B43] Wright SD, Weitz JI, Huang AJ, Levin SM, Silverstein SC, Loike JD (1988). Complement receptor type three (CD11b/CD18) of human polymorphonuclear leukocytes recognizes fibrinogen. Proc Natl Acad Sci USA.

[B44] Ishikawa H, Hirata S, Andoh Y, Kubo H, Nakagawa N, Nishibayashi Y, Mizuno K (1996). An immunohistochemical and immunoelectron microscopic study of adhesion molecules in synovial pannus formation in rheumatoid arthritis. Rheumatol Int.

[B45] Dejana E, Languino LR, Polentarutti N, Balconi G, Ryckewaert JJ, Larrieu MJ, Donati MB, Mantovani A, Marguerie G (1985). Interaction between fibrinogen and cultured endothelial cells. Induction of migration and specific binding. J Clin Invest.

[B46] Smiley ST, King JA, Hancock WW (2001). Fibrinogen stimulates macrophage chemokine secretion through toll-like receptor 4. J Immunol.

[B47] Clavel C, Nogueira L, Laurent L, Iobagiu C, Vincent C, Sebbag M, Serre G (2008). Induction of macrophage secretion of tumor necrosis factor alpha through Fcgamma receptor IIa engagement by rheumatoid arthritis-specific autoantibodies to citrullinated proteins complexed with fibrinogen. Arthritis Rheum.

[B48] Cantaert T, De Rycke L, Bongartz T, Matteson EL, Tak PP, Nicholas AP, Baeten D (2006). Citrullinated proteins in rheumatoid arthritis: crucial but not sufficient!. Arthritis Rheum.

[B49] Ji H, Gauguier D, Ohmura K, Gonzalez A, Duchatelle V, Danoy P, Garchon HJ, Degott C, Lathrop M, Benoist C, Mathis D (2001). Genetic influences on the end-stage effector phase of arthritis. J Exp Med.

[B50] Moreland LW, Schiff MH, Baumgartner SW, Tindall EA, Fleischmann RM, Bulpitt KJ, Weaver AL, Keystone EC, Furst DE, Mease PJ, Ruderman EM, Horwitz DA, Arkfeld DG, Garrison L, Burge DJ, Blosch CM, Lange ML, McDonnell ND, Weinblatt ME (1999). Etanercept therapy in rheumatoid arthritis. A randomized, controlled trial. Ann Intern Med.

[B51] Plenge RM, Seielstad M, Padyukov L, Lee AT, Remmers EF, Ding B, Liew A, Khalili H, Chandrasekaran A, Davies LR, Li W, Tan AK, Bonnard C, Ong RT, Thalamuthu A, Pettersson S, Liu C, Tian C, Chen WV, Carulli JP, Beckman EM, Altshuler D, Alfredsson L, Criswell LA, Amos CI, Seldin MF, Kastner DL, Klareskog L, Gregersen PK (2007). TRAF1-C5 as a risk locus for rheumatoid arthritis – a genomewide study. N Engl J Med.

[B52] Remmers EF, Plenge RM, Lee AT, Graham RR, Hom G, Behrens TW, de Bakker PI, Le JM, Lee HS, Batliwalla F, Li W, Masters SL, Booty MG, Carulli JP, Padyukov L, Alfredsson L, Klareskog L, Chen WV, Amos CI, Criswell LA, Seldin MF, Kastner DL, Gregersen PK (2007). STAT4 and the risk of rheumatoid arthritis and systemic lupus erythematosus. N Engl J Med.

[B53] Begovich AB, Carlton VE, Honigberg LA, Schrodi SJ, Chokkalingam AP, Alexander HC, Ardlie KG, Huang Q, Smith AM, Spoerke JM, Conn MT, Chang M, Chang SY, Saiki RK, Catanese JJ, Leong DU, Garcia VE, McAllister LB, Jeffery DA, Lee AT, Batliwalla F, Remmers E, Criswell LA, Seldin MF, Kastner DL, Amos CI, Sninsky JJ, Gregersen PK (2004). A missense single-nucleotide polymorphism in a gene encoding a protein tyrosine phosphatase (PTPN22) is associated with rheumatoid arthritis. Am J Hum Genet.

[B54] Eisen MB, Spellman PT, Brown PO, Botstein D (1998). Cluster analysis and display of genome-wide expression patterns. Proc Natl Acad Sci USA.

